# Obstructive azoospermia as an unusual complication associated with herniorrhaphy of an omphalocele: a case report

**DOI:** 10.1186/1752-1947-5-234

**Published:** 2011-06-25

**Authors:** Kazunari Tsuchihashi, Kazutoshi Okubo, Kentaro Ichioka, Takeshi Soda, Koji Yoshimura, Akihiro Kanematsu, Osamu Ogawa, Hiroyuki Nishiyama

**Affiliations:** 1 Department of Urology, Graduate School of Medicine, Kyoto University, 54 Shogoin Kawahara-cho, Sakyo-ku, Kyoto 606-8507, Japan

## Abstract

**Introduction:**

Iatrogenic damage to the seminal tract is one of the causes of obstructive azoospermia, which can be an indication for reconstruction surgery. We present a case of obstructive azoospermia as an unusual complication after neonatal herniorrhaphy of an omphalocele.

**Case presentation:**

A 30-year-old Japanese man was diagnosed with obstructive azoospermia. He had undergone herniorrhaphy of an omphalocele immediately after birth. Reconstruction surgery of both seminal tracts was performed to pursue the possibility of naturally achieved pregnancy. Intra-operative findings demonstrated that both vasa deferentia were interrupted at the internal inguinal rings, although the abdominal end of the right vas leading to the seminal vesicle was found in the abdominal cavity. The discharge from the stump of the testicular end had no sperm, although the right epididymal tubules were dilated with motile sperm. Therefore, we performed right-sided vasovasostomy in the internal inguinal ring and ipsilateral epididymovasostomy simultaneously.

**Conclusion:**

To the best of our knowledge, this is the first report describing obstructive azoospermia as an unusual complication of herniorrhaphy of an omphalocele. It is important to pay attention to the existence of seminal tracts in such surgery as well as in inguinal herniorrhaphy.

## Introduction

Obstructive azoospermia is one cause of male infertility. Obstruction of the seminal tracts can be caused by iatrogenic damage, by a congenital anomaly or by infectious diseases [1]. Although the most common iatrogenic cause is bilateral repair of an inguinal hernia, here we present a case of obstructive azoospermia caused by herniorrhaphy of an omphalocele immediately after birth. As far as we are aware, this is the first such report.

## Case presentation

**Figure 1:**
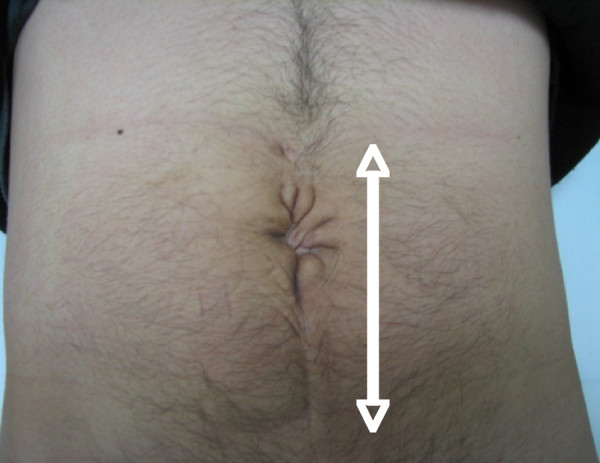
**Surgical scar from the omphalocele repair of the abdomen**. Note that the scar is far from the scrotum and the groin.

**Figure 2:**
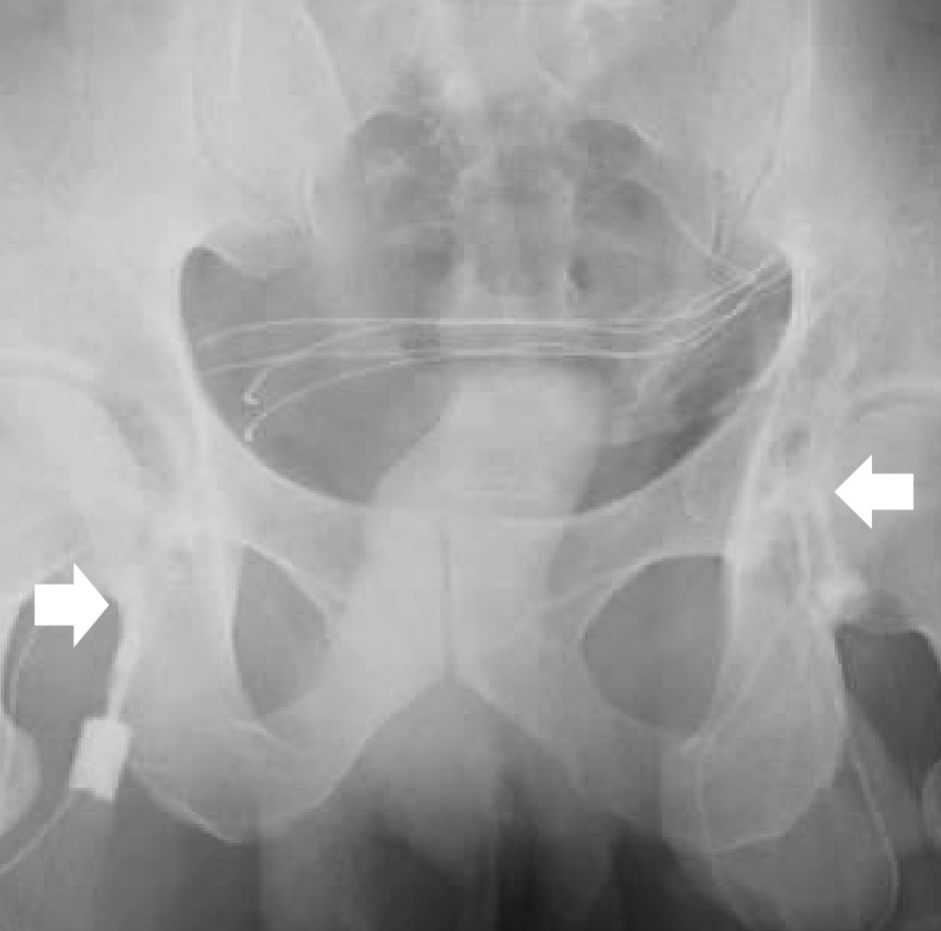
**Bilateral vasography in which contrast medium was injected from into the vas deferens on the epididymal side**. Both vasa deferentia were interrupted at the internal inguinal rings (arrows).

A 30-year-old Japanese man who had undergone herniorrhaphy for an omphalocele immediately after birth was referred to our hospital. He presented to our hospital with 1 year of infertility after his marriage. Multiple semen analyses revealed azoospermia, but a scrotal examination revealed no abnormalities in the testes or in the vas deferens or epididymis on both sides. Magnetic resonance imaging of the scrotum revealed no abnormal findings in the seminal vesicles, the prostate or the ejaculatory ducts. An endocrinological examination demonstrated that his serum follicle-stimulating hormone, luteinizing hormone and testosterone levels were within the normal range (4.1 mIU/ml, 3.6 mIU/ml and 7.48 ng/ml, respectively). The surgical scar from his omphalocele repair was found in the midline of the abdomen (Figure 1), far from the scrotum and the groin. He had no history of inguinal herniorrhaphy or vasectomy. A histopathological section of a right testicular biopsy revealed normal spermatogenesis (Johnsen score count 10) [2]. Following the pre-operative diagnosis of obstructive azoospermia, reconstructions of both seminal tracts were performed because the patient and his wife strongly wanted to pursue the possibility of a natural pregnancy. Intra-operative vasography demonstrated that both vasa deferentia were interrupted at the internal inguinal rings (Figure 2). The abdominal end of the left vas could not be identified, but the abdominal end of the right spermatic duct was found in the abdominal cavity. The discharge from the stump of the testicular end of the vas had no sperm, whereas the right epididymal tubules were dilated with motile spermatozoa. Therefore, we performed a right-sided vasovasostomy and ipsilateral epididymovasostomy simultaneously. Disappointingly, post-operative semen analyses demonstrated azoospermia. Finally, he fathered a child by intracytoplasmic sperm injection (ICSI) using testicular sperm that had been retrieved and cryopreserved at the time of the testicular biopsy.

## Discussion

Azoospermia is one of the causes of male infertility. Azoospermic patients whose testes show normal spermatogenesis on the basis of histopathology are diagnosed as having obstructive azoospermia. Cases of obstructive azoospermia are classified into two groups according to the obstruction sites. One is an obstruction in the vas deferens, and the other is one in the epididymis. The causes of epididymal obstruction include infectious diseases and congenital anomalies [1], and the causes of vasal obstruction include iatrogenic injury or congenital anomalies. The best-known causes of iatrogenic injury are vasectomy and bilateral inguinal herniorraphy [3]. There have been several reports of other causes of iatrogenic injury, including hydrocelectomy [4], appendectomy [3], spermatocelectomy [3] and renal transplantation [3]. However, to our knowledge, there has been no previous report in the literature on obstructive azoospermia after herniorrhaphy of an omphalocele. This is the first report showing that such surgery immediately after birth can cause azoospermia when the patient matures.

Omphalocele is a disease of neonates involving the herniation of intra-abdominal organs because of an abdominal wall defect, and emergency operations are generally required in most cases. The reported escaping organs include the small intestine, liver, bladder and ovary [5]. There is no report regarding the escape of seminal tracts; however, we think there is a possibility that the vas deferens can escape from the abdominal cavity when the defect in the abdominal wall is extensive. Our patient presented with azoospermia, but he had normal spermatogenesis and obstruction of both vasa deferentia at the internal inguinal rings. These findings are similar to those seen in cases of obstructive azoospermia after repair of bilateral inguinal hernias. It is important to pay attention to the seminal tracts during herniorrhaphy of an omphalocele, as well as in inguinal herniorrhaphy.

The vasa deferentia are very thin in neonates [6]. This might be why iatrogenic damage is often identified after pediatric surgery [3]. The second reason might be the recent advances in assisted reproductive technologies (ART). Because of the growth in the number of centers offering ART and the ability to perform *in vitro *fertilization (IVF) in cases of severe male factor infertility, there has been a trend toward pursuing IVF, including testicular sperm extraction ICSI, as a first-line therapy for obstructive azoospermia [7]. This trend might lead to the clinician's missing rare complications after hernia surgery. Thus, the need for long-term follow-up of all patients who underwent pediatric hernia surgery is emphasized.

## Conclusion

The case of our patient indicates that herniorrhaphy of an omphalocele can be an iatrogenic cause of obstructive azoospermia. This is the first report describing obstructive azoospermia as an unusual complication of this surgery. It is important to pay attention to the seminal tracts in herniorrhaphy of an omphalocele, as well as in inguinal herniorrhaphy.

## Consent

Written informed consent was obtained from the patient for publication of this case report and any accompanying images. A copy of the written consent is available for review by the Editor-in-Chief of this journal.

## Competing interests

The authors declare that they have no competing interests.

## Authors' contributions

KT, TS and HN performed the surgical procedure and reported the case. KY and AK interpreted and analyzed the findings. OO participated in the diagnostic and therapeutic decisions. KO and KI made major contributions to the writing of the manuscript. All authors read and approved the final manuscript.
